# Metabolic Regulation of Trisporic Acid on *Blakeslea trispora* Revealed by a GC-MS-Based Metabolomic Approach

**DOI:** 10.1371/journal.pone.0046110

**Published:** 2012-09-25

**Authors:** Jie Sun, Hao Li, Qipeng Yuan

**Affiliations:** State Key Laboratory of Chemical Resource Engineering, College of Life Science and Technology, Beijing University of Chemical Technology, Beijing, People’s Republic of China; University of Nottingham, United Kingdom

## Abstract

The zygomycete *Blakeslea trispora* is used commercially as natural source of â-carotene. Trisporic acid (TA) is secreted from the mycelium of *B. trispora* during mating between heterothallic strains and is considered as a mediator of the regulation of mating processes and an enhancer of carotene biosynthesis. Gas chromatography-mass spectrometry and multivariate analysis were employed to investigate TA-associated intracellular biochemical changes in *B. trispora*. By principal component analysis, the differential metabolites discriminating the control groups from the TA-treated groups were found, which were also confirmed by the subsequent hierarchical cluster analysis. The results indicate that TA is a global regulator and its main effects at the metabolic level are reflected on the content changes in several fatty acids, carbohydrates, and amino acids. The carbon metabolism and fatty acids synthesis are sensitive to TA addition. Glycerol, glutamine, and ã-aminobutyrate might play important roles in the regulation of TA. Complemented by two-dimensional electrophoresis, the results indicate that the actions of TA at the metabolic level involve multiple metabolic processes, such as glycolysis and the bypass of the classical tricarboxylic acid cycle. These results reveal that the metabolomics strategy is a powerful tool to gain insight into the mechanism of a microorganism’s cellular response to signal inducers at the metabolic level.

## Introduction

Carotenoids are tetraterpenoid organic pigments that are vital for human health and generally cannot be manufactured by animals. A variety of natural and synthetic carotenoids are available nowadays. Carotenoid production from microbial sources is receiving more attention because of public sensitivity regarding synthetic food additives [1]. The greatest yields of â-carotene have been obtained with a mixture of plus (+) and minus (−) strains of *Blakeslea trispora* [2]. *B. trispora* belongs to the order Mucorales within the class Zygomycetes and is the only mucoral fungus of this group used commercially to produce â-carotene and lycopene.

Trisporic acid (TA), a C-18 terpenoid, is secreted from the mycelium of *B. trispora* during mating between heterothallic strains [3]. The first step of TA biosynthesis is â-carotene cleavage to form the TA precursors [4], which then are mutually exchanged into the opposite mating type to be converted to TA [5]. The addition of TA extracted from mating cultures significantly increases carotene production in (−) strain cultures [6], [7]. The expression of the metabolites is associated with the maintenance, growth, and functions of the cell [8]. Some previous studies on *B. trispora* showed that the transcription of the genes involved in carotene biosynthesis, energy metabolism, cell wall synthesis, and regulatory processes were strongly induced during mating [9], [10], which might be regulated, in part, through TA. However, little direct evidence has been provided to demonstrate the mechanism of TA regulation in *B. trispora* at the metabolic level.

Gas chromatography-mass spectrometry (GC-MS)-based analytical methods have been successfully used to analyze metabolites [11] and screened for environmentally-induced metabolic changes in microbes [12]–[14]. Therefore, metabolomic analysis provides a powerful approach to investigate the metabolic responses to environmental or cellular changes. Moreover, metabolomics complements genomics, transcriptomics as well as proteomics and facilitates metabolic engineering towards designing superior biocatalysts and cell factories [15].

In the present study, the metabolites of *B. trispora* were analyzed using GC-MS and multivariate data analysis to demonstrate TA responses at the metabolic level. The metabolites contributing the differences between the control and TA-treated groups were found using principal components analysis (PCA) and confirmed by hierarchical cluster analysis (HCA). Two-dimensional electrophoresis (2-DE) was performed to complement the results of the metabolomic analysis. The results provide insight into the regulatory mechanisms of TA on *B. trispora* at the metabolic level and have the potential to improve carotene production in natural or gene engineered microbes.

## Materials and Methods

### Strains and Culture Conditions

*B. trispora* ATCC 14272 (−) was chosen for this study because (−) strains do not produce TA [16] and the production of â-carotene in (−) strains increased when exogenous TA was added to the culture medium [7]. *B. trispora* ATCC 14272 (-) was maintained on potato dextrose agar plates (30% (w/v) potato extract, 2% (w/v) glucose, 0.1% (w/v) KH_2_PO_4_, 0.01% (w/v) MgSO_4_). Spores were harvested by rinsing the mature cultures with distilled water. A total of 4×10^4^ spores were inoculated into 50 mL of liquid synthetic mucor medium (SMM, composed of glucose 40 g, asparagine 2 g, KH_2_PO_4_ 0.5 g, MgSO_4_ 0.25 g, thiamine 0.5 mg and 1 liter of distilled water [17] ) containing 1% (w/v) malt extract and 0.1% (v/v) Tween 20 in 250-mL shaker flasks. The flasks were shaken at 180 rpm in darkness at 28°C.

### Separation of TA

TA was extracted as previously described [18]. Briefly, TA recovered from the acidified (pH 2) culture medium was purified using silica gel thin-layer chromatography. The purified TA was resolved in ethanol. Approximate measurements of the TA concentration were calculated using the specific extinction coefficients for TA (E_325 nm 1% cm_ = 572) [19]. A Micromass 70-VSE mass spectrometer with an ion source temperature of 200°C and a probe temperature of 25°C was used to identify the preparative TA.

### Sampling, Quenching, and Extraction of Intracellular Metabolites

For the treatments, 50 µg of TA-B was added to the 36-h cultures (50 mL). And equal volume of ethanol without TA-B was added to the control. After culturing for 3, 6, and 12 h, respectively, the mycelia were filtered through a four-layer gauze, washed by pre-chilled water (4°C), and squeezed quickly to remove water. The culture was quenched in liquid nitrogen [20], followed by the extraction of the intracellular metabolites using pure methanol [21]. Briefly, *B. trispora* mycelia were homogenized in liquid nitrogen. Mycelia powder (∼300 mg) was transferred into 1.5 ml Eppendorf tube. Then 0.75 ml cold methanol (−40°C) was added. The mixture was vortexed rigorously for 30 s and centrifuged at 8000×g for 10 min at −4°C. The supernatant was collected and an additional 0.75 ml of pre-chilled pure methanol was added to the pellet. The mixture was vortexed for 30 s prior to centrifugation (8000×g, −4°C, 10 min). Both supernatants were pooled together and stored at −80°C until use. To correct the minor variations that occur during sample preparation and analysis, 15 µg/mL of adonitol was added as an internal standard. The pellet was dried and weighed to obtain the dry weight of the sampled cells.

### Data Acquisition by GC-MS

For the GC-MS analysis, sample derivatization was performed in accordance with the two-stage technique of Roessner et al., with minor variations [22]. Briefly, 0.6 ml of extraction was dried in a vacuum centrifuge dryer. 50 µL of 20 mg/mL methoxyamine hydrochloride in pyridine was added to the samples followed by 2-h incubation at 30°C. Subsequently, the samples were derivatized upon the addition of 50 µL MSTFA (N-methyl-N-trimethylsilyltrifluoroacetamide) and incubated for an additional hour at 37°C. The derivatized samples were stored at −40°C and equilibrated to room temperature prior to injection.

The GC-MS analysis was performed using a GC-MS QP2010 Plus instrument (Shimadzu, Kyoto, Japan). The derivatized samples (1 µL) were injected at a 1∶30 split ratio onto a DB-5 column (30 m×0.25 mm id with a 0.25 µm film thickness) (J & W Scientific, Folson, USA). During GC, helium was supplied at a constant rate of 3 mL/min. The temperature program started at 80°C for 1 min, and increased at a rate of 10°C/min until it reached 160°C, followed by temperature ramping at 5°C/min to a final temperature of 300°C, which was held constant for 6 min. A mass range of m/z 80–500 was scanned to confirm the retention times of target analytes. The interface temperature was maintained at 280°C. Four independent biological repeats were performed for the control and TA-treated groups at each time point. Measurements for each sample were performed in triplicate.

### Multivariate Data Analysis

Principal component analysis (PCA), a nonsupervised method, was performed for the GC-MS data using the SIMCA package (Umetrics, Umea, Sweden) to discern the control groups from the TA-treated groups. Only those areas of the peaks with a signal to noise ration higher that 3 were included in the analysis with the SIMCA-P software. The peak areas in each sample were normalized to the peak area of the internal standard adonitol on the same chromatograph. Differences between the samples could be detected in the PCA score plots, whereas the retention times of the peaks responsible for the differences could be observed in the corresponding PCA loading plots. A metabolite with a VIP value greater than 1 demonstrates a significant contribution to the separation of groups within PCA models [23]. The terms *R* ^2^X (cum) and *Q* ^2^ (cum) were used to evaluate the fit degree and the predictive ability of the PCA models.

The integrated peak areas of multiple derivative peaks belonging to the same compound were summed and considered as a single compound. The hierarchical cluster analysis (HCA) was used to differentiate the metabolites contributing to the differences between the control and TA-treated groups. The HCA was performed using Euclidian distances and complete linkage grouping with Cluster 3.0 (http://bonsai.ims.u-tokyo.ac.jp/~mdehoon/software/cluster/).

### Proteomic Analysis

The mycelia (∼400 mg) were collected at 12 h after the addition of TA and homogenized in 4 ml cold extraction buffer (20 mM Tris-Cl, pH 8.0, 0.5% (v/v) Triton X-100, 0.5 M EDTA, 10 mM β-mercaptoethanol, and 1 mM PMSF). The cell debris was removed by centrifugation at 12,000×g for 10 min at 4°C. Then, the watery solution below the carotene layer was removed carefully. The methanol/chloroform precipitation method [24] was employed to remove the nucleic acids and pigments. The protein extracts were resuspended in 130 µl rehydration buffer (7 M urea, 2 M thiourea, 4% (w/v) CHAPS, 0.2% (v/v) Bio-Lyte 3/10 ampholyte, 50 mM DTT, and 0.002% (w/v) bromophenol blue). Then 17 cm, pH 5–8 IPG strips (Bio-Rad, California, USA) were rehydrated for 14 h with 1.2 mg of protein in 400 µL rehydration buffer. The 2-DE and protein quantitative analysis was conducted according to Li et al. [25]. The data were analyzed with PDQuest software (version 8.0.1, Bio-Rad). The intensity of each protein spot was normalized to the sum of all spot volumes to obtain a relative volume. The Student’s t-test was used at a significance level of 95% for the statistical analysis of the gels, and 1.5 and 0.5 were chosen as the upper and lower limits, respectively. Only the spots showing statistically significant differences in protein abundance were considered as differentially expressed. Three independent biological repeats were performed for the control and TA-treated groups, with each biological repeat made up of two technical repeats at least.

The protein spots were manually excised from the gel and identified according to the method of Katayama et al. [26]. Mass spectrometric analysis was performed using a MALDI-TOF/TOF mass spectrometer 4800 (Applied Biosystems, Foster City, USA). Both the MS and MS/MS data were integrated and processed using the GPS Explorer V3.6 software (Applied Biosystems) with default parameters. All spectra were searched using the MASCOT V2.1 search engine (Matrix Science Ltd., London, UK). Due to the absence of protein and DNA databases containing information for *B. trispora*, a protein database was constructed using information obtained from *Phycomyces blakesleeanus*, *Mucor circinelloides*, and *Rhizopus oryzae* (protein sequences obtained from http://genome.jgi-psf.org/Phybl2/Phybl2.home.html, http://genome.jgi-psf.org/Mucci2/Mucci2.home.html, and http://www.broadinstitute.org/annotation/genome/rhizopus_oryzae/MultiHome.html). The following search parameters were used: trypsin as the digestion enzyme; one missed cleavage site; fixed modifications of carbamidomethyl (C), partial modifications of acetyl (Protein N-term) and oxidation (M); fixed modifications; 100 ppm for the precursor ion tolerance; and 0.3 Da for the fragment ion tolerance.

### Real Time PCR Analysis

Total RNA was extracted using TRIzol (Invitrogen, Carlsbad, USA). The cDNA synthesis reaction was performed using oligo(dT)_18_ and MMLV reverse transcriptase (Promega, Madison, USA) according to the manufacturer’s instructions. Degenerate PCR primers for the EST of genes were designed based on the conserved sequences among *P. blakesleeanus*, *M. circinelloides*, and *R. oryzae* obtained from the websites indicated above, with consideration for the codon usage of *B. trispora* (http://www.kazusa.or.jp/codon/) (**Table S1**). The six PCR fragments were gel purified and sequenced.

The real-time PCR primers used in this study are presented in **Table S2**. The primers were designed according to the EST sequences obtained above. The first-strand cDNA synthesis was performed using 1 µg of total RNA and 0.4 µg of random hexamers. The relative quantification was performed using the RealSuper Mixture (CWBiotech, Beijing, China) on a Mastercycler Realplex2 cycler (Eppendorf, Hamburg, Germany). Three biological repeats were performed for the control and TA-treated groups. The measurements of each sample were performed in triplicate. The results were normalized to the *tef1* gene and presented as relative to the expression of the corresponding group without TA addition (value = 1) using the comparative method of Livak and Schmittgen [27].

## Results

### Differential Metabolites in Response to TA Treatment

TA-B was isolated and identified using silica gel thin-layer chromatography and mass spectrometry, respectively (**Figure S1**). Then metabolic profiles of *B. trispora* were detected after TA addition using GC-MS. A gradient temperature program was adopted to obtain the total ion chromatogram (TIC) with higher chromatographic resolution. Representative GC-MS TICs for the metabolites are shown in **Figure S2**. Most of the peaks in the chromatograms were identified as amino acids, organic acids, carbohydrates, and fatty acids using the NIST mass spectral library.

**Figure 1:**
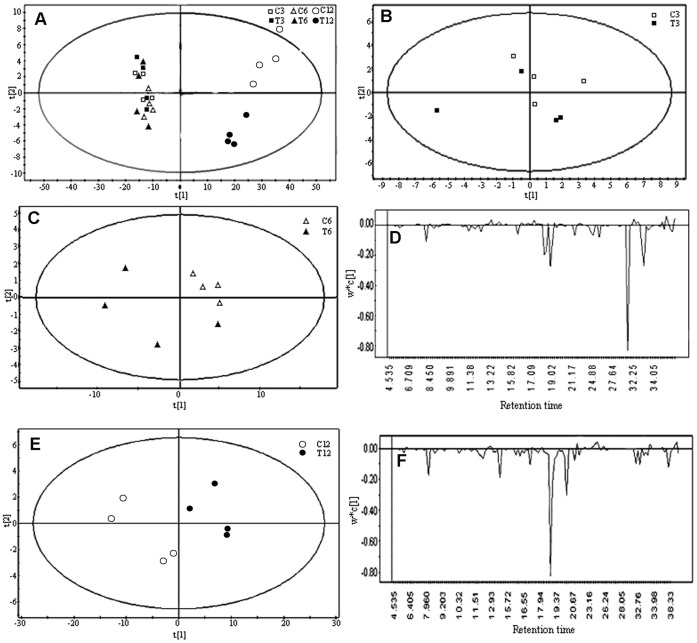
PCA to compare the metabolome of the control and TA-treated groups using GC-MS. (A) Score plot of all samples from the control and TA-treated groups at 3, 6, and 12 h after TA addition; (B) Score plot for the control and TA-treated groups at 3 h after TA addition; (C and D) Score and loading plots for the control and TA-treated groups at 6 h after TA addition; (E and F) Scores and loading plots for the control and TA-treated groups at 12 h after TA addition. In the score plot, the confidence interval was defined using the Hotelling’s T2 ellipse (95% confidence interval). T3, T6, and T12 represent the samples at 3, 6, and 12 h after TA addition; C3, C6, and C12 represent the samples at the corresponding control group.

PCA is a linear transformation of the multiple variables into a lower dimensional space which retain maximal amount of information about the variables. Each principle component is a linear combination of the variables for an observation or a sample. To obtain a comprehensive comparison of the metabolic profiles of the control and TA-treated groups, the PCA score plots of the first two principal components (t [1], t [2]) were employed (**Figure 1**). The PCA analysis showed that all samples from the control and TA-treated groups at 3, 6, and 12 h after TA addition were not separated along PC1 and PC2 (**Figure 1A**). The two groups were not separated (**Figure 1B**) at 3 h, suggesting the similar metabolic profiles between these two groups. However, the PCA models were constructed successfully at 6 and 12 h, and the two groups were separated (**Figure 1C and E**). When the two groups were separated with four principal components at 6 h after TA addition, *R* ^2^X (cum) = 0.968 and *Q* ^2^ (cum) = 0.753. When the two groups were separated with two principal components at 12 h after TA addition, *R* ^2^X (cum) = 0.960 and *Q* ^2^ (cum) = 0.821. The major metabolic perturbations causing clusters at 6 and 12 h were identified from the PCA loadings plots (**Figure 1D and F**).

**Figure 2:**
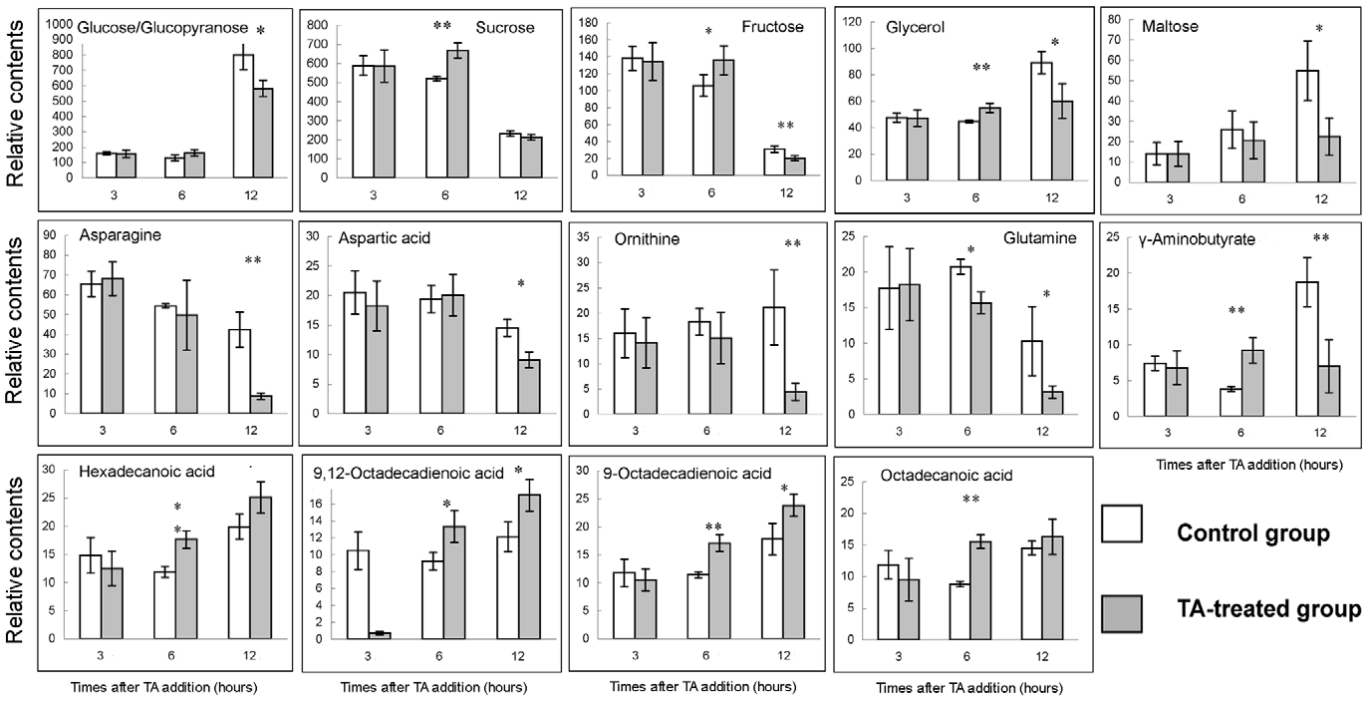
Relative contents of differential metabolites for the control and TA-treated groups at 3, 6, and 12 h after TA addition. *(*P*<0.05) and **(*P*<0.01). The relative contents of each metabolite were expressed as the ratio of its peak areas to that of the internal standard adonitol on the same chromatograph.

The VIP plots demonstrate that some of identified metabolites contribute to the group classification. We identified the peaks (variable importance value >1) from the VIP plots of 6 and 12 h (**Figure S3**), respectively, which were responsible for the differences between the control and TA-treated groups. Peak areas of multiple derivative peaks belonging to the same compound were summed. Only the contents of the differential metabolites found by PCA were analyzed below. The relative contents of these differential metabolites are shown in **Figure 2**. As compared with the control group, *B. trispora* contained higher levels of sucrose, fructose, glycerol, γ-aminobutyrate (GABA), 9-octadecadienoic acid, octadecanoic acid, hexadecanoic acid, and 9, 12-octadecadienoic acid at 6 h after TA addition (*P*<0.05). The contents of glucose, maltose, glycerol, GABA, asparagines, aspartic acid, ornithine, and glutamine at 12 h after TA addition were lower than that in the corresponding control (*P*<0.05). Moreover, the levels of 9-octadecadienoic acid, hexadecanoic acid, and 9, 12-octadecadienoic acid were still higher at 12 h after TA addition than that in the corresponding control (*P*<0.05).

**Figure 3:**
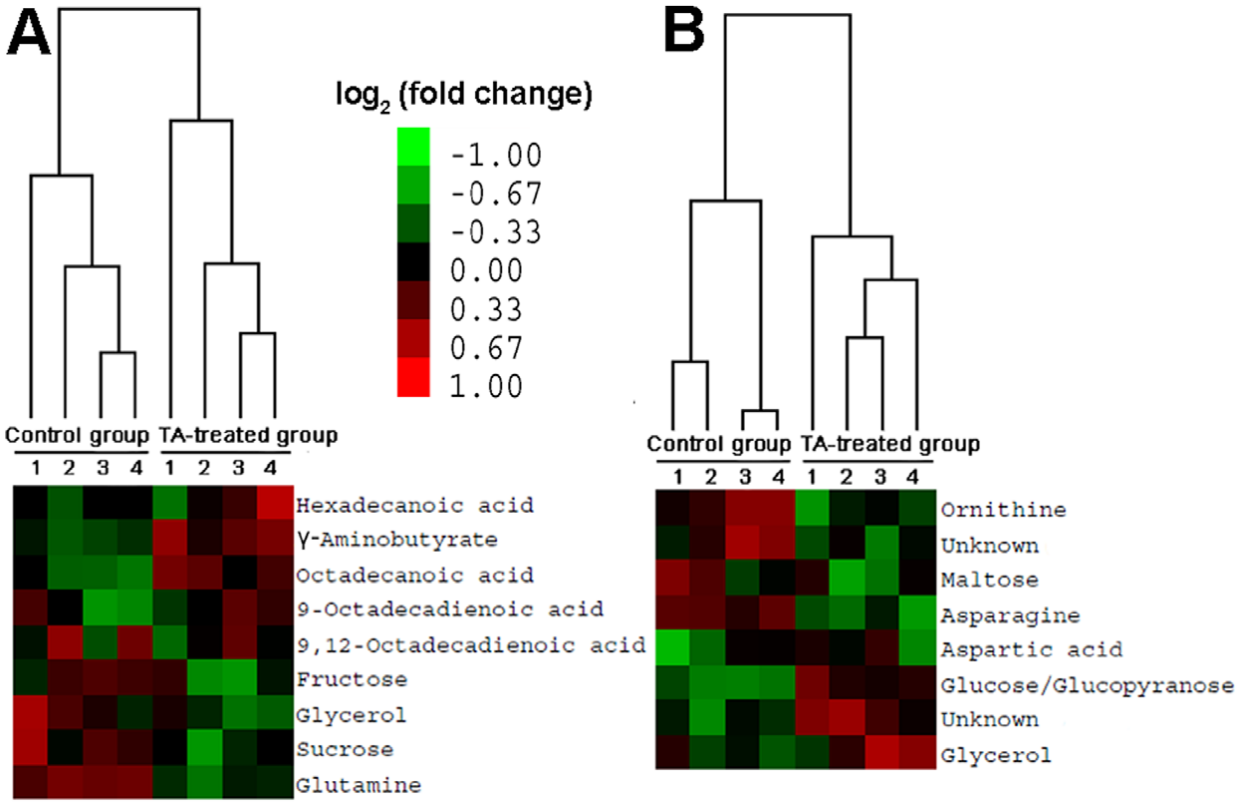
Comparison of the differential metabolites in *B. trispora* using HCA between the control and TA-treated groups. (A) Differential metabolites present at 6 h after TA addition; (B) Differential metabolites, including two unidentified metabolites, present at 12 h after TA addition. The Arabic numerals 1,2,3,4 are referring to the biological repeats in the same group.

To confirm the differential metabolites found by PCA, HCA analysis was performed using Cluster 3.0. Log base 2 is used to calculate the fold changes of the metabolite levels. As shown in **Figure 3**, the fold changes are represented by different colors. The samples with similar metabolic profiling are clustered into one group. The results showed that the control and TA-treated groups were successfully clustered at 6 and 12 h after TA addition, respectively (**Figure 3**). The results of the HCA further confirmed the metabolites that contributed to the differences between the control and TA-treated groups.

### Differential Proteins in Response to TA Treatment

**Figure 4:**
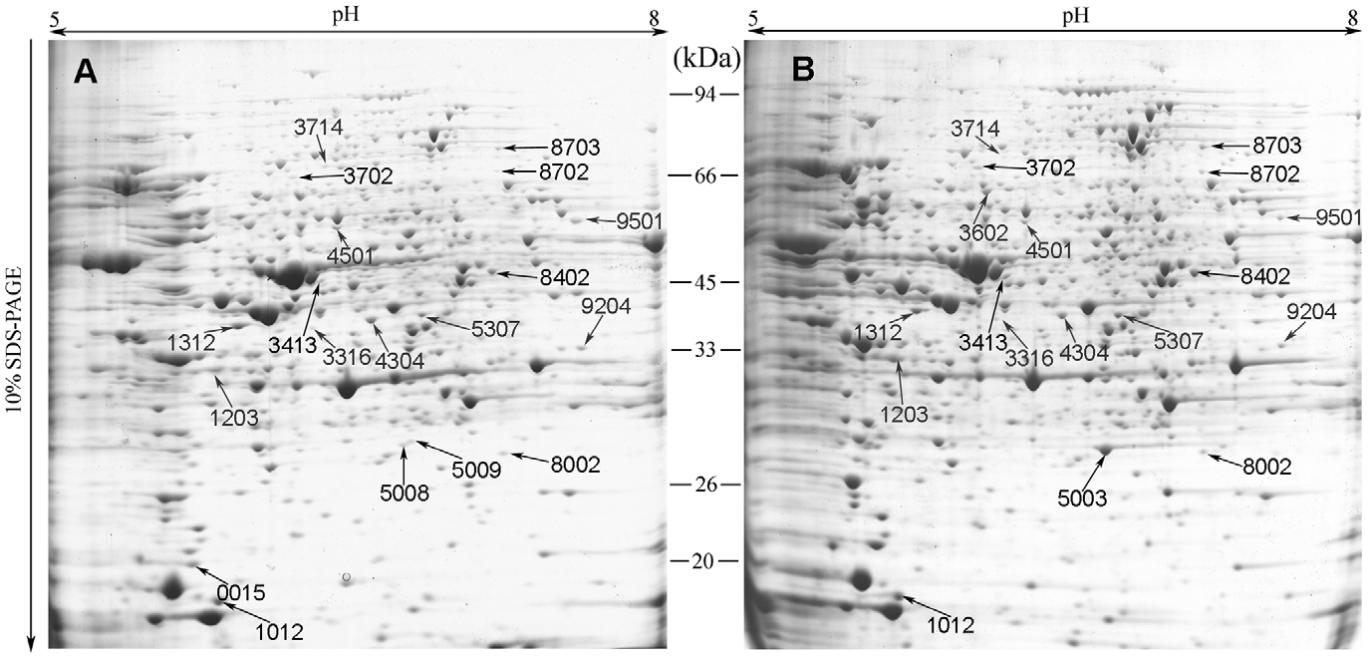
2-D maps of the colloidal Coomassie-stained proteins from *B. trispora* in the control (**A**) **or the TA-treated** (**B**) **cultures.** The proteins were initially separated on an IPG strip (pH 5–8; 17 cm) followed by separation in the second dimension on a 10% gel. The proteins in the TA-treated group that exhibited a significant change in expression (≤0.5-fold or ≥1.5-fold, *P*<0.05) as compared with the control group are labeled in the figures.

To complement the results of the metabolomic analysis, a 2-DE analysis of the total protein in *B. trispora* was conducted after a 12-h TA treatment. Subsequently, we identified a series of proteins with changes in their expression levels (**Figure 4**). A total of 21 protein spots showed a significant change (*P*<0.05) in intensity of less than 0.5-fold or more than 1.5-fold between the control and TA-treated groups. Of the proteins detected, 7 were up-regulated and 14 were down-regulated as a result of TA addition.

The identified proteins are listed in **Table S3**. These differentially expressed proteins participate in a variety of cellular functions, including the transport and metabolism of nucleotides, amino acids, carbohydrates, lipids, and coenzymes, energy production and conversion, posttranslational modification, protein turnover, and translation. The results indicate that these physiological processes might play crucial roles in the responses of *B. trispora* to TA.

### Real Time PCR Analysis of Differentially Expressed Proteins

**Figure 5:**
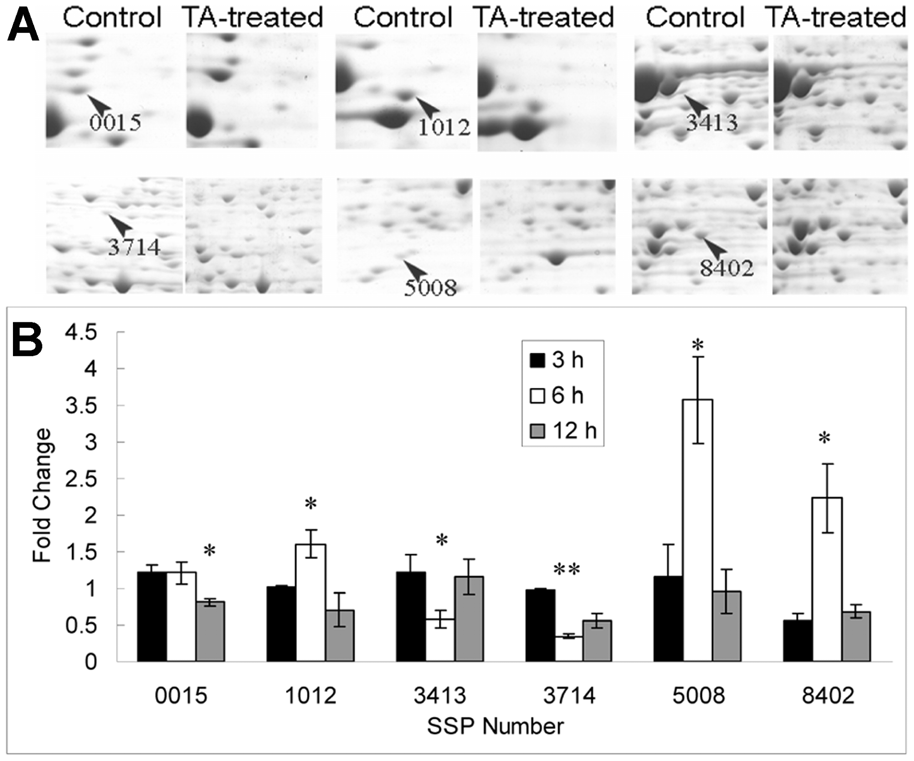
Expression patterns of several proteins at the protein and transcriptional levels. (A) The detailed expression patterns of several spots in the control and TA-treated groups at 12 h after TA addition; (B) Time courses of the transcription of proteins in (A) between the control and TA-treated groups at 3, 6, and 12 h after TA addition;. *(*P*<0.05) and **(*P*<0.01). SSP 0015, adenine phosphoribosyltransferase; SSP 1012, cytochrome c oxidase, subunit Vb; SSP 3413, diphosphomevalonate decarboxylase; SSP 3714, aconitase/homoaconitase; SSP 5008, proteasome beta type 7; SSP 8402, adenylosuccinate synthetase.

The expression patterns of six identified proteins at the protein and transcriptional levels are shown in **Figure 5**. The results showed that the proteins observed, except for adenine phosphoribosyltransferase (SSP 0015), had significantly different transcriptional levels at 6 h after TA addition (*P*<0.05), which might result from the time-gap between transcription and translation. The transcriptional levels of cytochrome c oxidase subunit Vb (SSP 1012), proteasome beta type 7 (SSP 5008), and adenylosuccinate synthetase (SSP 8402) were significantly increased after TA addition (*P*<0.05), and the levels of adenine phosphoribosyltransferase (SSP 0015), diphosphomevalonate decarboxylase (SSP 3413), and aconitase/homoaconitase (SSP 3714) were decreased (*P*<0.05). The transcriptional levels of adenine phosphoribosyltransferase (SSP 0015), aconitase/homoaconitase (SSP 3714), and adenylosuccinate synthetase (SSP 8402) were associated with the amount of their translation products after TA addition, while the transcriptional levels of the other proteins did not correspond with the amounts of their translation products. Thus, the mRNA and protein levels are not well correlated, indicating that post-transcriptional regulation might be involved in the TA responses.

## Discussion

Carotenoids are widely used as antioxidant to reduce cellular or tissue damage, stimulator for the immune system, and colouring agent for food products. *B. trispora* accumulates â-carotene as the main carotene and is used on an industrial scale to produce â-carotene. â-Carotene production in *B. trispora* is greatly stimulated by TA. However, the metabolic regulation of TA on carotene accumulation in *B. trispora* remains unknown. To understand the effects of TA at the metabolic level, metabolites of *B. trispora* were analyzed using metabolomic approach in the present study. GC-MS was used to detected intracellular metabolites from the control and TA-treated groups. However, the sensitivity of GC-MS is not sufficient to detect intracellular trace trisporoids. The differential metabolites between the control and TA-treated groups were extracted from the PCA model. The major effects of TA are reflected on the content changes of these differential metabolites, including fatty acids, carbohydrates, and amino acids. The metabolites with little contribution to the differences between the control and TA-treated groups are not focused in this study and not discussed below. The differential metabolites were further confirmed by HCA.

2D-PAGE, as a useful technique to find differentially expressed proteins, was used to complement the results of metabolomic analysis. However, the differentially expressed enzymes involved in â-carotene synthesis, except for diphosphomevalonate decarboxylase, were not found in this study. The possible reasons might be involved in the narrow pH range of IPG strip used and the quantity of the 2D-PAGE map. In addition, the protein dots that did not meet the requirements about statistics and fold change were also not identified. Even so, 21 differentially expressed proteins involved in multiple cellular functions were still identified.

### The Effects of TA on Carotene and Fatty Acid Metabolism

The differential metabolites and proteins identified in this study were related to multiple metabolic pathways and physiological functions. Rao and Modi (1977) found that the addition of TA to the culture medium resulted in a four-fold increase in carotene production in the (−) strain of *B. trispora* [6]. In this study, the level of diphosphomevalonate decarboxylase (SSP 3413), which is involved in carotene biosynthesis, was increased 2.89-fold following a 12-h TA treatment (**Table S3** and **Figure 5A**). The addition of TA to the culture medium also increased the contents of fatty acids (9, 12-octadecadienoic acid, 9-octadecadienoic acid, octadecanoic acid, and hexadecanoic acid) in the mycelia at 6 h after TA addition (**Figure 2**). 3-Hydroxyacyl-CoA dehydrogenase (SSP 5009) catalyzes the third step of beta-oxidation in the catabolism of fatty acids; significantly decreased levels of this enzyme facilitated the accumulation of fatty acids induced after a 12-h incubation with TA (**Table S3**). The positive and significant correlation between the contents of â-carotene and 9-octadecadienoic acid was also observed in *Dunaliella salina* [28]. One possible reason of the positive correlation between the contents of â-carotene and fatty acids is that the biosynthesis of fatty acids and carotene both uses acetyl-CoA as the building unit. Therefore, TA might induce the accumulation of fatty acids and carotene by improving the biosynthesis of acetyl-CoA. In addition, the stimulation of oils containing primarily 9,12-octadecadienoic acid and 9-octadecadienoic acid on the production of â-carotene in *B. trispora* was observed [29], which might be related to the feedback inhibition of fatty acid synthesis and thus facilitate the transformation of more acetyl-CoA to â-carotene.

### The Effects of TA on Carbohydrate Metabolism

The synthesis of fatty acids and carotenes requires the use of NADPH, which is generated mainly from the pentose phosphate pathway (PPP). The PPP also converts glucose 6-phosphate to fructose 6-phosphate and glyceraldehyde 3-phosphate, which are further transformed to fructose and glycerol. Therefore, the PPP might participate in the accumulation of fructose and glycerol in the mycelia after 6 h of TA treatment. Following a 12-h incubation with TA, the glucose, fructose, and maltose contents in the mycelia decreased significantly (**Figure 2**), indicating the activation of glycolysis. Notably, the expression of phosphoglucomutase (SSP 3602), which catalyzes the conversion of glucose 6-phosphate to glucose 1-phosphate, was inhibited upon TA addition (**Table S3**). Therefore, when the glucose concentration was reduced, glycerol was utilized as a carbon source. Mantzouridou et al. observed that â-carotene was accumulated in *B. trispora* in higher amounts when both glucose and glycerol were added to the medium as compared with the use of glucose as the sole carbon source [30]. Therefore, we hypothesize that the catabolism of glycerol might be related to the increased carotene and fatty acids levels observed after TA addition.

**Figure 6:**
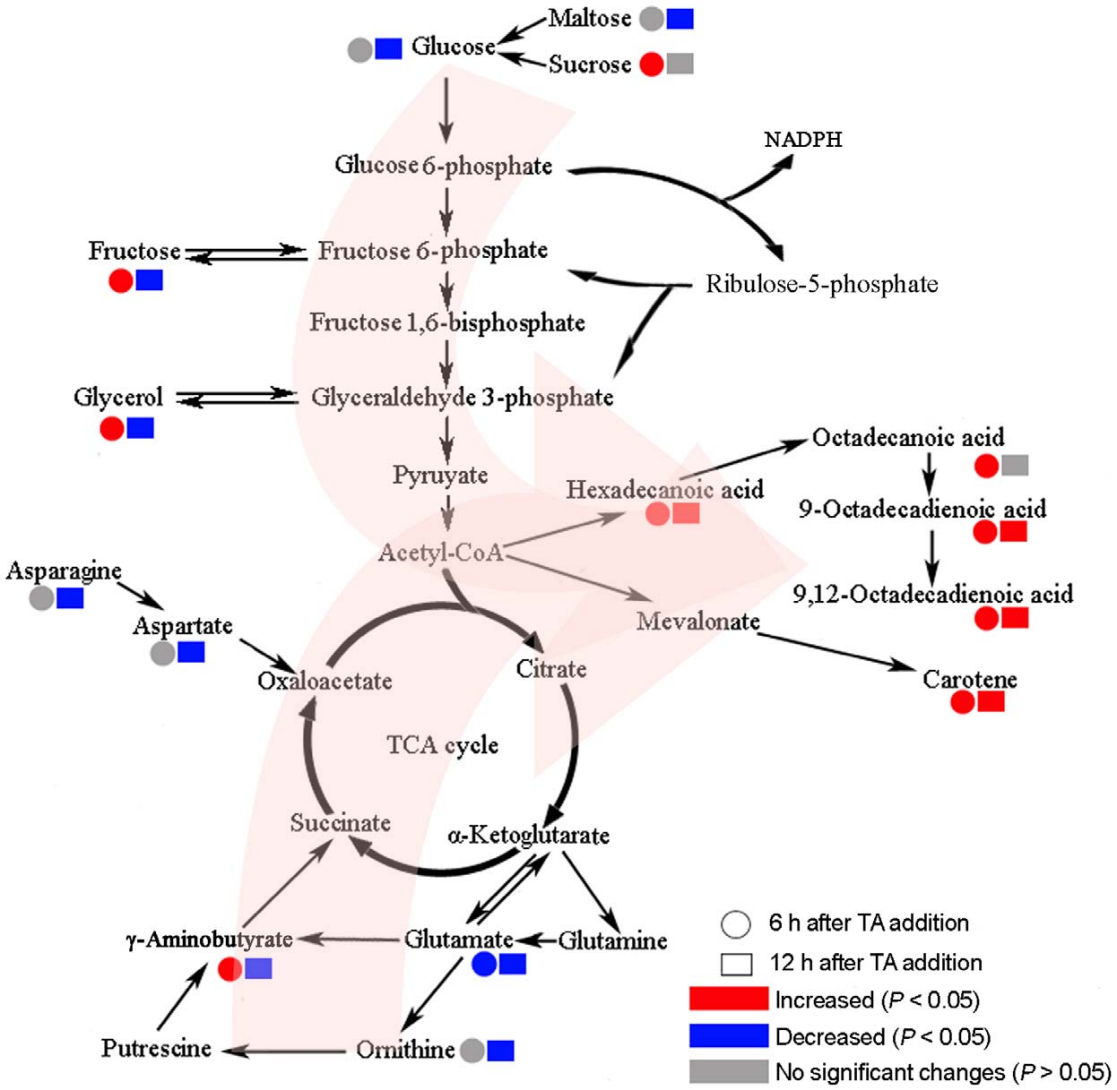
Scheme summarizing the responses to TA at the metabolic level in *B. trispora*. The pink arrows indicate the speculative changes in metabolic flux after TA addition.

### The Effects of TA on Amino Acid Metabolism

The GC-MS analysis showed decreased levels of amino acids at 12 h after TA addition, such as alanine, asparagine, aspartic acid, glutamine, proline, serine, threonine, and valine (**Figure 2**, some data were not shown). The amino acids participate in the TCA cycle and the biosynthesis of protein. Valine participates in the TCA cycle with the catalysis of the branched-chain amino acid transaminase as the first step. The level of branched-chain amino acid transaminase (SSP 4304) was decreased at 12 h after TA addition (**Table S3**). We also observed that the level of threonyl-tRNA synthetase (SSP 8703) was increased and ubiquitin-protein ligase (SSP 4501) was decreased (**Table S3**). However, to delineate the connection between TA and protein metabolism still needs further investigation.

The glutamine contents were lower than the corresponding control group at 6 and 12 h following TA addition in this study (**Figure 2**). Glutamine synthetase catalyzes the condensation of glutamic acid and ammonia to form glutamine and plays an essential role in the metabolism of nitrogen. TA treatment decreased the level of glutamine synthetase (SSP 1312) (**Table S3**), suggesting the conversion from glutamic acid to other metabolites. Ornithine is synthesized from acetyl-CoA and glutamic acid and converted to putrescine [31]. Putrescine is further converted to GABA, which is also synthesized from the decarboxylation of glutamic acid and converted to succinic acid. GABA metabolism plays multiple roles in nitrogen and carbon metabolism, sporulation, differentiation, and development [32]. In this study, the GABA contents were increased after 6 h and decreased after 12 h following TA addition (**Figure 2**). Whether GABA is involved in TA regulation in *B. trispora* still needs further study. The asparagine, aspartic acid, and ornithine contents did not change significantly at 6 h after TA addition; however, the asparagine, aspartic acid, and ornithine contents were decreased at 12 h after TA addition (**Figure 2**), suggesting that the TCA cycle bypass, comprising á-ketoglutaric acid, glutamine, ornithing, putresine, GABA, and succinic acid, might be involved in the metabolic regulation of TA at 12 h after TA addition.

In addition, the increased level of adenylosuccinate synthetase (SSP 8402) and the decreased level of adenine phosphoribosyltransferase (SSP 0015) shown by 2-DE experiment (**Table S3**) could both facilitate the production of AMP. AMP can be converted to ADP and ATP or used to activate protein kinases. AMP synthesis might be involved in TA responses.

The pathways and differential metabolites described above are shown in **Figure 6**. The levels of metabolites, such as glucose, frucose, glycose, asparagine, and glutamate, which “input” various pathways, were decreased, especially at 12 h after TA addition. However, the levels of fatty acids and carotene, as “output metabolites” from metabolic pathways, were increased. Therefore, from **Figure 6**, TA seems to facilitate the metabolic flux from glycolysis (especially glycerol metabolism), the pentose phosphate pathway, the TCA cycle towards the fatty acid and carotene synthesis. In addition, engineered eukaryotic microbes, such as *Saccharomyces cerevisiae* [33], *Candida utilis* [34], and *Neurospora crassa* [35], enabled production of carotene by the introduction of carotene biosynthetic pathways. According to the speculations above, the operations for the inhibition of fatty acids biosynthesis and the TCA cycle and the improvement of the glycolysis pathway (especially the glycerol metabolism) and the pentose phosphate pathway might further improve the flux and energy forwards carotene synthesis in *B. trispora* and engineered eukaryotic microbes. And we will proceed to further confirmation of the regulatory manner at the metabolic level in carotene-producing microbes.

In summary, the effects of TA at the metabolic level are primarily reflected in the metabolism of fatty acids, carbohydrates, and amino acids. The metabolism of carbohydrates and fatty acids is more sensitive to TA than the metabolism of amino acids. The differential metabolites glycerol, glutamine, and GABA might participate in the regulatory process of TA, but their roles need further investigation. Hence, TA is a global regulator and its regulation involves multiple metabolic pathways. These results also show that the metabolomics strategy is a powerful tool to gain insight into the microorganism’s cellular responses to signal inducers.

## Supporting Information

Figure S1 **Thin layer chromatography of** ***B. trispora*** **(+/-) pH 2 culture (A) and mass spectrum of TA-B (B).**
(TIF)

Figure S2 **Representative GC-MS total ion chromatograms of the samples from the control and TA-treated groups after chemical derivatization.** T3, T6, and T12 represent the samples at 3, 6, and 12 h after TA addition; C3, C6, and C12 represent the samples at the corresponding control group.
(TIF)

Figure S3 **Variable importance of the projection plots for the intracellular metabolites from the control and TA treated groups at 6 (A) and 12 h (B) after TA addition.**
(TIF)

Table S1 **Degenerate PCR primers used in this study.**
(DOC)

Table S2 **Real-time PCR primers used in this study.**
(DOC)

Table S3 **Differentially expressed proteins identified by MS/MS.**
(DOC)

## References

[1] Bhosale P (2004) Environmental and cultural stimulants in the production of carotenoids from microorganisms. Appl Microbiol Biotechnol 63: 351–361. 14566431 10.1007/s00253-003-1441-1

[2] Lopez-Nieto MJ, Costa J, Peiro E, Mendez E, Rodriguez-Saiz M, et al (2004) Biotechnological lycopene production by mated fermentation of *Blakeslea trispora* . Appl Microbiol Biotechnol 66: 153–159. 15248041 10.1007/s00253-004-1669-4

[3] van den Ende H, Werkman BA, van den Briel ML (1972) Trisporic acid synthesis in mated cultures of the fungus Blakeslea trispora. Arch Mikrobiol 86: 175–184. 5081117 10.1007/BF00413370

[4] Burmester A, Richter M, Schultze K, Voelz K, Schachtschabel D, et al (2007) Cleavage of beta-carotene as the first step in sexual hormone synthesis in zygomycetes is mediated by a trisporic acid regulated beta-carotene oxygenase. Fungal Genet Biol 44: 1096–1108. 17822929 10.1016/j.fgb.2007.07.008

[5] Schachtschabel D, David A, Menzel KD, Schimek C, Wöstemeyer J, et al (2008) Cooperative biosynthesis of Trisporoids by the (+) and (−) mating types of the zygomycete *Blakeslea trispora* . Chembiochem 9: 3004–3012. 19035372 10.1002/cbic.200800477

[6] Rao S MV (1977) Carotenogenesis: Possible mechanism of action of trisporic acid in *Blakeslea trispora* . Cellular and Molecular Life Sciences 33: 31–33.

[7] van den Ende H (1968) Relationship between sexuality and carotene synthesis in *Blakeslea trispora* . J Bacteriol 96: 1298–1303. 5686001 10.1128/jb.96.4.1298-1303.1968 PMC252448

[8] Beecher CWW (2003) The human metabolome. In: Harrigan GG, Goodacre R (eds) Metabolic profiling: its role in biomarker discovery and gene function analysis. Kluwer, Boston, 311–319.

[9] Schmidt AD, Heinekamp T, Matuschek M, Liebmann B, Bollschweiler C, et al (2005) Analysis of mating-dependent transcription of *Blakeslea trispora* carotenoid biosynthesis genes carB and carRA by quantitative real-time PCR. Appl Microbiol Biotechnol 67: 549–555. 15744487 10.1007/s00253-005-1941-2

[10] Kuzina V, Ramirez-Medina H, Visser H, van Ooyen AJ, Cerda-Olmedo E, et al (2008) Genes involved in carotene synthesis and mating in *Blakeslea trispora* . Current genetics 54: 143–152. 18677485 10.1007/s00294-008-0206-x

[11] Koek MM, Muilwijk B, van der Werf MJ, Hankemeier T (2006) Microbial metabolomics with gas chromatography/mass spectrometry. Anal Chem 78: 1272–1281. 16478122 10.1021/ac051683+

[12] Strelkov S, von Elstermann M, Schomburg D (2004) Comprehensive analysis of metabolites in Corynebacterium glutamicum by gas chromatography/mass spectrometry. Biol Chem 385: 853–861. 15493881 10.1515/BC.2004.111

[13] Barsch A, Patschkowski T, Niehaus K (2004) Comprehensive metabolite profiling of *Sinorhizobium meliloti* using gas chromatography-mass spectrometry. Funct Integr Genomics 4: 219–230. 15372312 10.1007/s10142-004-0117-y

[14] Stephanopoulos G, Alper H, Moxley J (2004) Exploiting biological complexity for strain improvement through systems biology. Nat Biotechnol 22: 1261–1267. 15470466 10.1038/nbt1016

[15] Mashego MR, Rumbold K, De Mey M, Vandamme E, Soetaert W, et al (2007) Microbial metabolomics: past, present and future methodologies. Biotechnol Lett 29: 1–16. 17091378 10.1007/s10529-006-9218-0

[16] Sutter RP, Capage DA, Harrison TL, Keen WA (1973) Trisporic acid biosynthesis in separate plus and minus cultures of *Blakeslea trispora*: identification by Mucor assay of two mating-type-specific components. J Bacteriol 114: 1074–1082. 4712567 10.1128/jb.114.3.1074-1082.1973 PMC285367

[17] Hesseltine CW, Anderson RF (1957) Microbiological production of carotenoids. I. Zygospores and carotene produced by intraspecific and interspecific crosses of Choanephoraceae in liquid media. Myeologia 49: 449.

[18] Schimek C, Kleppe K, Saleem AR, Voigt K, Burmester A, et al (2003) Sexual reactions in Mortierellales are mediated by the trisporic acid system. Mycol Res 107: 736–747. 12951800 10.1017/s0953756203007949

[19] Nieuwenhuis M (1975) Sex specificity of hormone synthesis in Mucor mucedo. Arch Microbiol 102: 167–169. 1115561 10.1007/BF00428363

[20] Hajjaj H BP, Goma G, François J (1998) Sampling techniques and comparative extraction procedures for quantitative determination of intra-and extracellular metabolites in filamentous fungi. FEMS Microbiol Lett 164: 195–200.

[21] Villas-Bôas SG, Hojer-Pedersen J, Akesson M, Smedsgaard J, Nielsen J (2005) Global metabolite analysis of yeast: evaluation of sample preparation methods. Yeast (Chichester, England) 22: 1155–1169. 10.1002/yea.1308 16240456

[22] Roessner U, Luedemann A, Brust D, Fiehn O, Linke T, et al (2001) Metabolic profiling allows comprehensive phenotyping of genetically or environmentally modified plant systems. Plant Cell 13: 11–29. 11158526 10.1105/tpc.13.1.11 PMC2652711

[23] Szeto SS, Reinke SN, Sykes BD, Lemire BD (2010) Mutations in the *Saccharomyces cerevisiae* succinate dehydrogenase result in distinct metabolic phenotypes revealed through (1)H NMR-based metabolic footprinting. J Proteome Res 9: 6729–6739. 20964315 10.1021/pr100880y

[24] Wessel D, Flugge UI (1984) A method for the quantitative recovery of protein in dilute solution in the presence of detergents and lipids. Anal Biochem 138: 141–143. 6731838 10.1016/0003-2697(84)90782-6

[25] Li Q, Huang J, Liu S, Li J, Yang X, et al (2011) Proteomic analysis of young leaves at three developmental stages in an albino tea cultivar. Proteome science 9: 44. 21806834 10.1186/1477-5956-9-44 PMC3162873

[26] Katayama H, Nagasu T, Oda Y (2001) Improvement of in-gel digestion protocol for peptide mass fingerprinting by matrix-assisted laser desorption/ionization time-of-flight mass spectrometry. Rapid Commun Mass Spectrom 15: 1416–1421. 11507753 10.1002/rcm.379

[27] Livak KJ, Schmittgen TD (2001) Analysis of relative gene expression data using real-time quantitative PCR and the 2^-ΔΔCT^ method. Methods 25: 402–408. 11846609 10.1006/meth.2001.1262

[28] Mendoza H, Martel A, Jiménez del Río M, G GR (1999) Oleic acid is the main fatty acid related with carotenogenesis in *Dunaliella salina* . J Appl Psychol 11: 15–19.

[29] Ciegler A, Arnold M, Anderson RF (1959) Microbiological production of carotenoids. V. Effect of lipids and related substances on production of beta-carotene. Appl Microbiol 7: 98–101. 13637892 10.1128/am.7.2.98-101.1959 PMC1057476

[30] Mantzouridou F, Naziri E, Tsimidou MZ (2008) Industrial glycerol as a supplementary carbon source in the production of beta-carotene by *Blakeslea trispora* . J Agric Food Chem 56: 2668–2675. 18370396 10.1021/jf703667d

[31] Boyle SM, Markham GD, Hafner EW, Wright JM, Tabor H, et al (1984) Expression of the cloned genes encoding the putrescine biosynthetic enzymes and methionine adenosyltransferase of *Escherichia coli* (speA, speB, speC and metK). Gene 30: 129–136. 6392022 10.1016/0378-1119(84)90113-6

[32] Kumar S, Punekar NS (1997) The metabolism of 4-aminobutyrate (GABA) in fungi. Mycol Res 101: 403–409.

[33] Verwaal R, Wang J, Meijnen JP, Visser H, Sandmann G, et al (2007) High-level production of beta-carotene in *Saccharomyces cerevisiae* by successive transformation with carotenogenic genes from *Xanthophyllomyces dendrorhous* . Appl Environ Microbiol 73: 4342–4350. 17496128 10.1128/AEM.02759-06 PMC1932764

[34] Shimada H, Kondo K, Fraser PD, Miura Y, Saito T, et al (1998) Increased carotenoid production by the food yeast *Candida utilis* through metabolic engineering of the isoprenoid pathway. Appl Environ Microbiol 64: 2676–2680. 9647847 10.1128/aem.64.7.2676-2680.1998 PMC106443

[35] Wang GY, Keasling JD (2002) Amplification of HMG-CoA reductase production enhances carotenoid accumulation in *Neurospora crassa* . Metab Eng 4: 193–201. 12616689 10.1006/mben.2002.0225

