# Supplementary figures and images for: Metabolic Regulation of Trisporic Acid on Blakeslea trispora Revealed by a GC-MS-Based Metabolomic Approach
Source: PLoS One. 2012 Sep 25;7(9):e46110. doi: 10.1371/journal.pone.0046110 (PMC3457941; doi:10.1371/journal.pone.0046110)

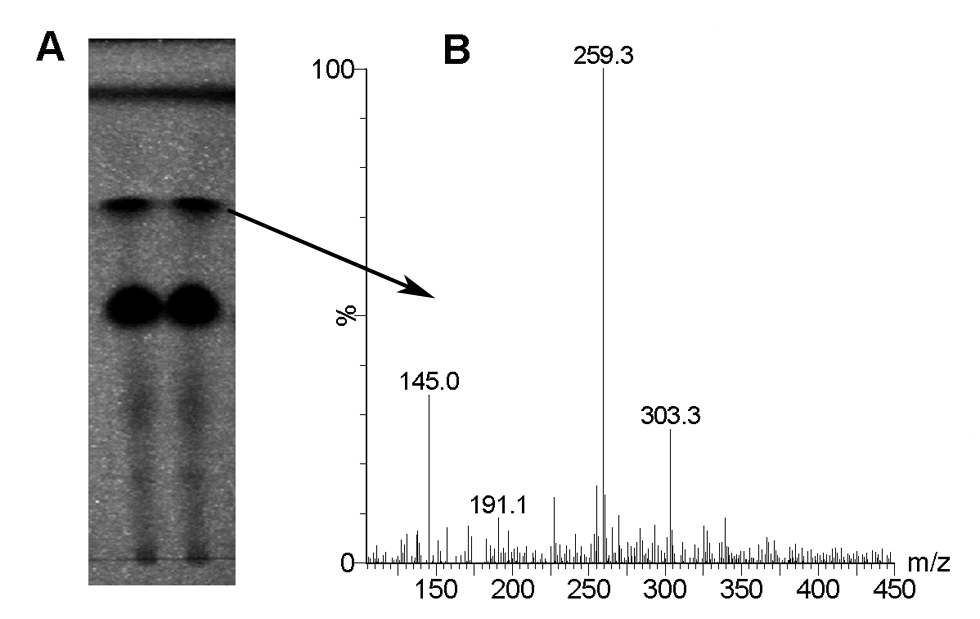

Supplement: Figure S1 — Thin layer chromatography of B. trispora (+/-) pH 2 culture (A) and mass spectrum of TA-B (B). (TIF) [file pone.0046110.s001.tif]

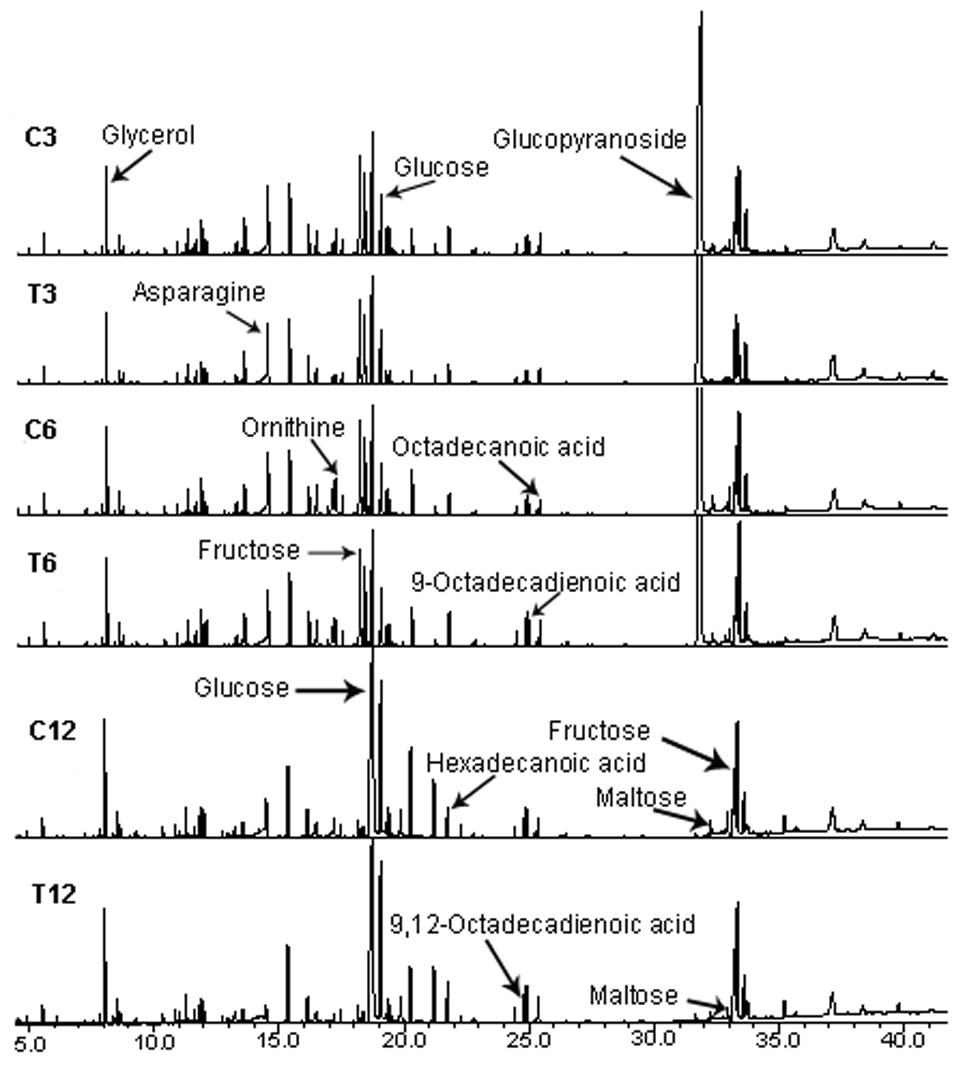

Supplement: Figure S2 — Representative GC-MS total ion chromatograms of the samples from the control and TA-treated groups after chemical derivatization. T3, T6, and T12 represent the samples at 3, 6, and 12 h after TA addition; C3, C6, and C12 represent the samples at the corresponding control group. (TIF) [file pone.0046110.s002.tif]

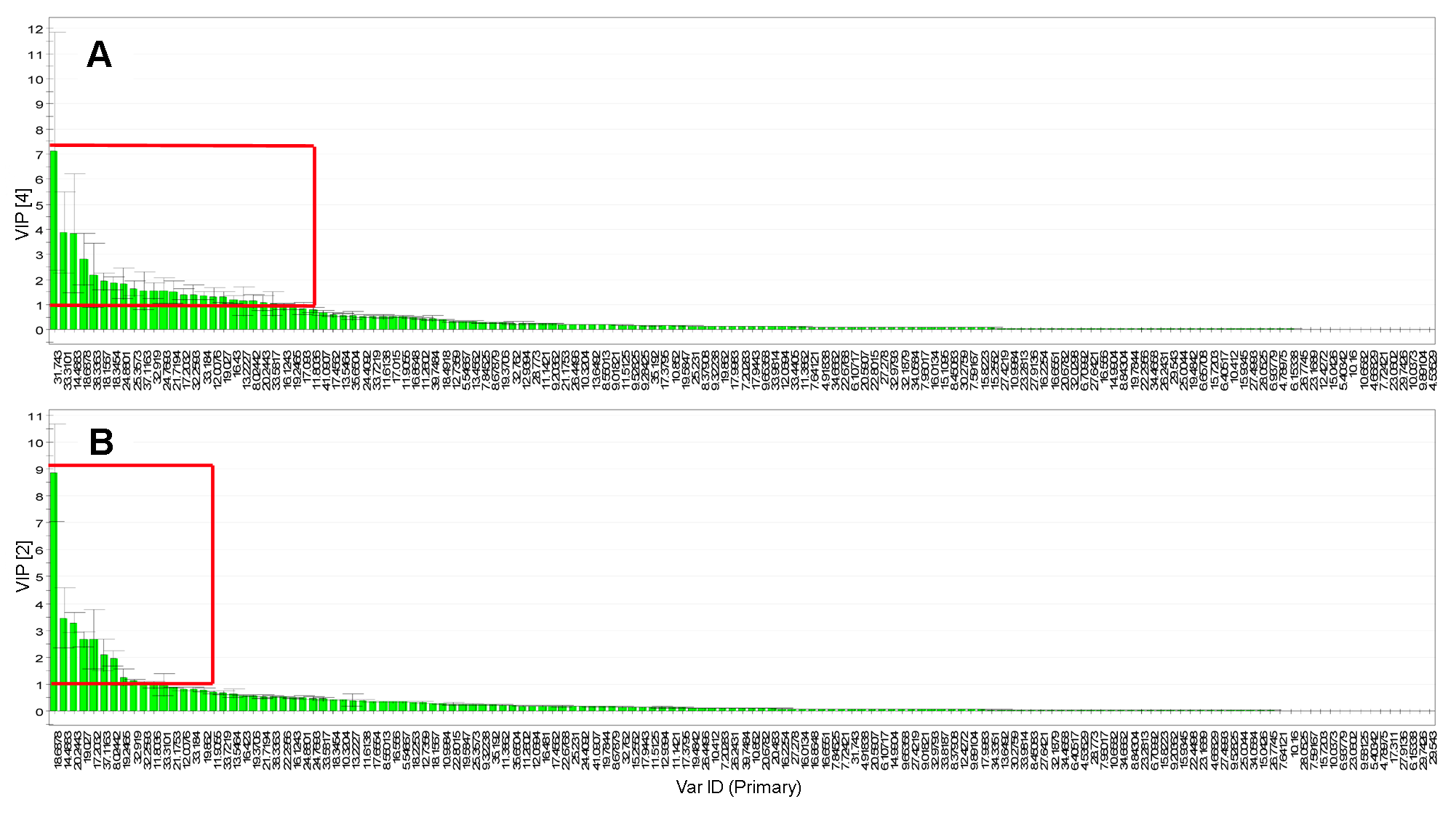

Supplement: Figure S3 — Variable importance of the projection plots for the intracellular metabolites from the control and TA treated groups at 6 (A) and 12 h (B) after TA addition. (TIF) [file pone.0046110.s003.tif]
