# Supplementary material for: Metabolic Regulation of Trisporic Acid on Blakeslea trispora Revealed by a GC-MS-Based Metabolomic Approach
Source: PLoS One. 2012 Sep 25;7(9):e46110. doi: 10.1371/journal.pone.0046110 (PMC3457941; doi:10.1371/journal.pone.0046110)
Supplement: Table S1 — Degenerate PCR primers used in this study. (DOC) [file pone.0046110.s004.doc]

**Table S**1

| **SSP No.** | **Forward primers (5′→3′)** | **Reverse primers (5′→3′)** |
| --- | --- | --- |
| 0015 | CAACYWCGACATTGAACGCATC | CASCAGARCCACCAGTRGCAATC |
| 1012 | ATGTTTGCTCTTCGTCGTGC | TTARTGGTGGTGHTCHTCNTCA |
| 3413 | AAGAACARRCGHATGCACAACTG | CCAGGATAGCCTTCTTCATNSCYTCCA |
| 5008 | AGATCCATTAWATTGCHCCCAACAT | ACACTCATWGCWGCCAATGAAC |
| 8402 | TACTCHTCCAAGGCCTCHCG | AACCAACCRCAACGACGCTT |
| 3714 | GGTGCHACNACCTCCATGTTCC | GTGATGATNGAGTTCTTGTCACC |

**Degenerate PCR primers used in this study**
