# Supplementary material for: Metabolic Regulation of Trisporic Acid on Blakeslea trispora Revealed by a GC-MS-Based Metabolomic Approach
Source: PLoS One. 2012 Sep 25;7(9):e46110. doi: 10.1371/journal.pone.0046110 (PMC3457941; doi:10.1371/journal.pone.0046110)
Supplement: Table S2 — Real-time PCR primers used in this study. (DOC) [file pone.0046110.s005.doc]

**Table S2**

| **SSP No.** | **GenBank accession No.** | **Forward primers (5′→3′)** | **Reverse primers (5′→3′)** |
| --- | --- | --- | --- |
| 0015 | JK818422 | GTTTCTTGTTTGGTCCTTTGATCG | CCGTATTCCTTAACGTAGGTGGC |
| 1012 | JK818423 | AAGCTCCAAGGCAAGGAACTCTT | ATAACATCGTGGGATTCAACAGGA |
| 3413 | JK818424 | TGCTTCCGTGAGACTCGTGCTA | GGACAAAGGTTCAATGGGGCTAC |
| 5008 | JK818425 | GACAGCCATGACATTATTAAAGCAGA | CAGTAGCATCAAATCCACCCAAGA |
| 8402 | JK818426 | TGCTCGTTTCAGAAGTATGGTCG | TTTGTTCGTGAAGATAAGGGATGG |
| 3714 | JK818427 | CATCAACCAAGCCAACGAACAC | GCGAGAACAATACCACCAGCGT |
| *tef1* | AF157235 | CTTCTCAAGCCGATTGTGCTATTC | GGACACCCAAGGTGAAGGCAA |

**Real-time PCR primers used in this study**
