# Supplementary material for: Metabolic Regulation of Trisporic Acid on Blakeslea trispora Revealed by a GC-MS-Based Metabolomic Approach
Source: PLoS One. 2012 Sep 25;7(9):e46110. doi: 10.1371/journal.pone.0046110 (PMC3457941; doi:10.1371/journal.pone.0046110)
Supplement: Table S3 — Differentially expressed proteins identified by MS/MS. (DOC) [file pone.0046110.s006.doc]

**Table S3**

| **KOG class ^a^** | **SSP No.** | **Protein name ^b^** | **Speices** | **Protein id ^c^** | **Theo Mr/PI ^d^** | **Exp Mr/PI ^e^** | **MudPIT Score ^f^** | **Peptides matched ^g^** | **Fold change (TA-treated/Control)** |
| --- | --- | --- | --- | --- | --- | --- | --- | --- | --- |
| Nucleotide transport and metabolism | 0015 | adenine phosphoribosyltransferase | *R. oryzae* | RO3G_02497 | 19.56/5.80 | 19.86/5.70 | 143 | 6 | 0.00 |
|  | 3316 | 3'(2'),5'-bisphosphate nucleotidase | *M. circinelloides* | 156693 | 36.56/5.69 | 39.81/6.17 | 296 | 7 | 0.38 |
|  | 8402 | Adenylosuccinate synthetase | *M. circinelloides* | 168799 | 47.89/6.29 | 46.23/7.21 | 63 | 8 | 2.04 |
| Amino acid transport and metabolism | 1312 | Glutamine synthetase | *M. circinelloides* | 154694 | 25.05/5.79 | 41.04/5.90 | 151 | 3 | 0.46 |
|  | 4304 | Branched-chain-amino-acid transaminase | *M. circinelloides* | 156209 | 33.01/8.69 | 40.47/6.52 | 160 | 7 | 0.42 |
| Coenzyme transport and metabolism | 1203 | pyridoxine biosynthesis protein | *R. oryzae* | RO3G_11214 | 33.48/5.79 | 35.49/5.81 | 228 | 7 | 0.40 |
| Lipid transport and metabolism | 3413 | Diphosphomevalonate decarboxylase | *M. circinelloides* | 154844 | 45.48/5.86 | 45.5/6.20 | 46 | 4 | 2.89 |
| Carbohydrate transport and metabolism | 3602 | Phosphoglucomutase | *M. circinelloides* | 176399 | 61.28/5.51 | 70.82/6.13 | 213 | 8 | 0.30 |
| Secondary metabolites biosynthesis, transport and catabolism | 5009 | 3-hydroxyacyl-CoA dehydrogenase | *M. circinelloides* | 114253 | 27.05/5.24 | 27.66/6.74 | 34 | 2 | 0.01 |
| Energy production and conversion | 1012 | Cytochrome c oxidase, subunit Vb | *M. circinelloides* | 156398 | 16.54/5.81 | 16.5/5.85 | 113 | 2 | 0.44 |
|  | 3714 | Aconitase/homoaconitase | *P. blakesleeanus* | 74487 | 85.58/6.00 | 85.00/6.30 | 115 | 7 | 0.12 |
|  | 5003 | Mitochondrial FAD carrier protein | *R. oryzae* | RO3G_11059 | 23.98/9.63 | 27.16/6.75 | 33 | 1 | 463.74 |
|  | 9204 | NADH:ubiquinone oxidoreductase, 39kDa subunit | *M. circinelloides* | 90548 | 42.51/6.73 | 37.1/7.70 | 89 | 4 | 0.44 |
|  | 9501 | Aldehyde dehydrogenase | *M. circinelloide* | 151287 | 56.63/6.65 | 61.81/7.69 | 145 | 2 | 0.35 |
| Posttranslational modification and protein turnover | 5008 | proteasome beta type 7 | *R. oryzae* | RO3G_00008 | 29.10/6.69 | 27.35/6.58 | 27 | 2 | 0.01 |
|  | 8002 | proteasome beta type 4 | *R. oryzae* | RO3G_17028 | 30.19/6.26 | 27.11/7.25 | 37 | 3 | 2.08 |
| Translation, ribosomal structure and biogenesis | 8703 | Threonyl-tRNA synthetase | *R. oryzae* | RO3G_06614 | 82.04/6.56 | 89.75/7.25 | 155 | 5 | 3.15 |
| Cytoskeleton | 3702 | Ca2+-binding actin-bundling protein (fimbrin/plastin) | *M. circinelloides* | 135428 | 70.03/5.44 | 80.74/6.11 | 49 | 3 | 1.68 |
| Poorly Characterized | 4501 | Ubiquitin-protein ligase | *M. circinelloides* | 84363 | 54.58/6.14 | 60.5/6.34 | 111 | 5 | 0.50 |
|  | 5307 | RNA recognition motif, RNP-1 | *M. circinelloides* | 164874 | 36.79/6.74 | 40.54/6.82 | 39 | 2 | 0.30 |
|  | 8702 | ATP-binding cassette transporters | *M. circinelloides* | 149573 | 63.65/5.76 | 78.79/7.25 | 53 | 4 | 3.47 |

**Differentially expressed proteins identified by MS/MS**

^a^ Classifications of eukaryotic orthologous genes obtained from http://genome.jgi-psf.org/.

^b, c^ Protein name and id obtained from the database described in “Materials and Methods”.

^d^ Theoretical mass (kDa) and pI of identified proteins calculated from http://web.expasy.org/compute_pi/.

^e^ Experimental mass (kDa) and pI of identified proteins.

^f^ As calculated by MS-Fit software. Individual ions scores > 27 indicate identity or extensive homology (p<0.05).

^g^ Number of peptides matched.
